# Does field of view matter? Assessing its impact on Plus disease diagnosis in retinopathy of prematurity

**DOI:** 10.1186/s40942-025-00750-w

**Published:** 2025-11-21

**Authors:** Sayed Mehran Sharafi, Mobina Amanollahi, Nazanin Ebrahimiadib, Mohammad Hossein Nowroozzadeh, Marjan Imani Fooladi, Fatemeh Bazvand, Nader Mohammadi, Mohammad Bagher Rajabi, Hamid Riazi-Esfahani, Elias Khalili Pour

**Affiliations:** 1 Translational Ophthalmology Research Center, Farabi Eye Hospital, Tehran University of Medical Sciences, Tehran, Iran; 2 Ophthalmology Department, College of Medicine, University of Florida, Gainesville, FL USA; 3 Poostchi Ophthalmology Research Center, Department of Ophthalmology, School of Medicine, Shiraz University of Medical Sciences, Shiraz, Iran; 4 Clinical Pediatric Ophthalmology Department, UPMC, Children’s Hospital of Pittsburgh, Pittsburgh, USA; 5 Retinopathy of Prematurity Department, Farabi Eye Hospital, Tehran University of Medical Sciences, South Kargar Street, Qazvin Square, Qazvin Street, Tehran, Iran

**Keywords:** Plus disease, Retinopathy of prematurity, Retinal imaging, Computer-based image analysis, Deep learning

## Abstract

**Purpose:**

This study investigates the impact of field of view (FOV) on the diagnosis of Plus disease in retinopathy of prematurity (ROP), focusing on how different FOVs influence expert assessments.

**Methods:**

Fundus images from 91 ROP infants were captured using a RetCam, each including the optic disk (OD) and a ridge/demarcation line. Cropped versions with a radius of three disc diameters (3DD) centered on the OD were automatically extracted using a computer-based algorithm. Images were categorized into five (normal, pre-Plus, Plus1, Plus2, Plus3), three (non-Plus, pre-Plus, Plus), or two groups (non-Plus, Plus). Five experts graded both entire-view and 3DD images. Inter-expert reliability was assessed using Cronbach’s alpha.

**Results:**

Seventy-four 3DD and 77 entire-view images were graded. Cronbach’s alpha values were 0.933 (entire-view) and 0.942 (3DD) in the 5-level grading system, indicating slightly higher consistency among experts for 3DD images. Notable diagnostic shifts were observed when comparing entire-view and 3DD images, with 25.0% of Plus1 diagnoses shifting to pre-Plus and 16.9% of pre-Plus diagnoses shifting to normal in the 5-level grading system. Two experts showed substantial changes in their grades, with one expert upscaling 33.7% and downscaling 16.9% of diagnoses, and the other upscaling 21.7% and downscaling 15.7% when assessing entire-view compared to 3DD images.

**Conclusions:**

FOV significantly affects the diagnosis of Plus disease, with peripheral retinal features influencing expert judgments. Restricting the FOV to a 3DD area improved inter-expert reliability but led to notable diagnostic shifts. These findings highlight the need for standardized imaging protocols and the potential role of AI in reducing diagnostic variability in ROP.

## Introduction

ROP is a potentially blinding eye disorder that primarily happens in premature infants undergoing oxygen therapy. It is a major global cause of vision loss in the pediatric population [1]. It is characterized by abnormal development of retinal blood vessels, and the destructive neovascularization, can lead to retinal detachment and vision loss if not properly managed [2]. “Plus disease” and pre-Plus disease are terms used to categorize the severity of ROP based on the appearance of retinal blood vessels within zone I. These conditions represent a spectrum of vascular abnormalities, progressing from normal to pre-Plus to Plus disease. Plus disease signifies severe ROP, characterized by prominent tortuosity and dilation of the posterior retinal vessels. In contrast, pre-Plus disease describes retinal vessels that exhibit abnormal tortuosity and/or dilation, although not to the extent observed in plus disease [3]. The presence of Plus disease signifies a more aggressive progression of ROP and necessitates prompt intervention to prevent adverse outcomes [4]. Hence, accurate diagnosis of Plus disease is critical for timely and appropriate treatment of ROP. However, the diagnosis is inherently subjective and prone to inter-expert variability [5, 6]. This variability can lead to diagnostic biases, where experts may provide different assessments of the same retinal images. For instance, the multicenter Cryotherapy for ROP (Cryo-ROP) trial, involved trained experts in ROP diagnosis. Notably, 12% of the eyes diagnosed with threshold disease (defined as at least five contiguous or eight cumulative clock-hours of stage 3 ROP in zone I or II with Plus disease) by one expert, were diagnosed as pre-threshold (defined as any stage of ROP in zone I, stage 2 ROP with Plus disease in zone II, or any stage 3 ROP in zone II) during confirmatory examination by a second unmasked expert [7]. Similarly, another study was performed using 34 wide-angle retinal photographs from infants with ROP, being assessed by 22 experts. Upon 3-level grading (Plus, pre-Plus, neither), the invited experts agreed on the same diagnosis in merely four images. Also, using a 2-level grading system (Pus, non-Plus), the experts agreed on the same diagnosis in seven images [8]. Such biases can result in inconsistent treatment decisions, potentially affecting patient outcome [3].

A proposed source of diagnostic bias may stem from the variability in specialists’ fields of view when analyzing retinal images, perhaps leading to a focus on differing vascular features [6, 9]. The hypothesis suggests that different fields of view can influence the assessment of the severity of Plus disease. Specifically, peripheral pathologies such as ridge lines or vessel proliferation, which are not direct markers of Plus disease, might affect the expert’s overall focus and judgment.

The present study aims to analyze variations in the experts’ diagnoses between two fields of view, including (1) the entire view of wide-angle posterior retinal images and (2) cropped versions with a radius of three discs diameter centered on the optic disc (OD). A 3-disc diameter (3DD) area provides a consistent and easily definable region for automated image analysis and cropping, ensuring standardization across images. This area also aligns with the clinical focus of Plus disease assessment, which primarily centers on the posterior pole. This study explores whether or not the inclusion of peripheral retinal pathologies influences the diagnosis of Plus disease.

## Methods

### Study design and ethical considerations

This retrospective study was conducted at Farabi Eye Hospital and adhered to the guidelines specified in the Declaration of Helsinki. It was approved by the institutional review committee at the Tehran University of Medical Sciences **(IR.TUMS. FARABIH.REC.1403.039)** [10]. Written informed consent was obtained from the parents of the infants included in the study, allowing for imaging and participation in the research. All private information related to the patients was removed before establishing the image dataset to ensure that their identity and privacy would be protected.

### Subjects, image preparation, and grading routine

#### Subjects

Ninety-one infants were roughly equal male and female infants included in the study who had: (1) an average birth weight (BW) of 1305 gr with a standard deviation of 427 gr, (2) an average gestational age (GA) of 29.3 with a standard deviation of three weeks, (3) been evaluated at a ROP care unit at Farabi Eye Hospital in Tehran, Iran between May 2016 and April 2020, (4) who met the national criteria for ROP screening examination. Based on a published guideline, the criteria for screening of ROP in Iran, included infants with a BW less than 2000 g. Infants with (1) prior treatment for ROP and (2) other ocular or systemic conditions (i.e. bronchopulmonary dysplasia or respiratory distress syndrome) that could impact the retinal examinations, were excluded.

#### ROP experts

Five retina specialists with 6 to 20 years of experience were invited to carry out the grading of the fundus images.

#### Images

Ninety-one wide-angle posterior retinal images (RetCam; Clarity Medical Systems, Pleasanton, CA) were included from 91 preterm infants during standard clinical procedures. The size of the images was 1200 pixels by 1600 pixels. Each image had an OD and a ridge or demarcation line.

#### Creating 3DD version and cropping process

A 3DD zone offers a uniform and clearly defined area for automatic image cropping and guarantees consistency among all images included in the current study. This area also aligns with the clinical focus of Plus disease assessment, which primarily centers on zone I (the most posterior part of the retina, defined by a circle with a radius twice the distance from the OD center to the foveal center) [11]. 3DD versions of the images were automatically created by focusing on a distance of three disk diameters from the OD’s center and cropping out the rest of the images.

Given that OD detection is an important step in automating retinal image analysis, we earlier developed a Fast R-CNN object detector [12] based on a deep convolutional neural network (CNN) to identify the ODs. For this purpose, OD areas in 497 retinal images were annotated by an expert using a graphical user interface. Annotated images were augmented to 1988 images through geometrical transformations including horizontal, vertical, and diagonal rotations applied on the images and the corresponding bounding boxes around their ODs. Obtained images were resized by a scale of 0.3 and then utilized to train our Fast R-CNN object detector.

**Fig. 1:**
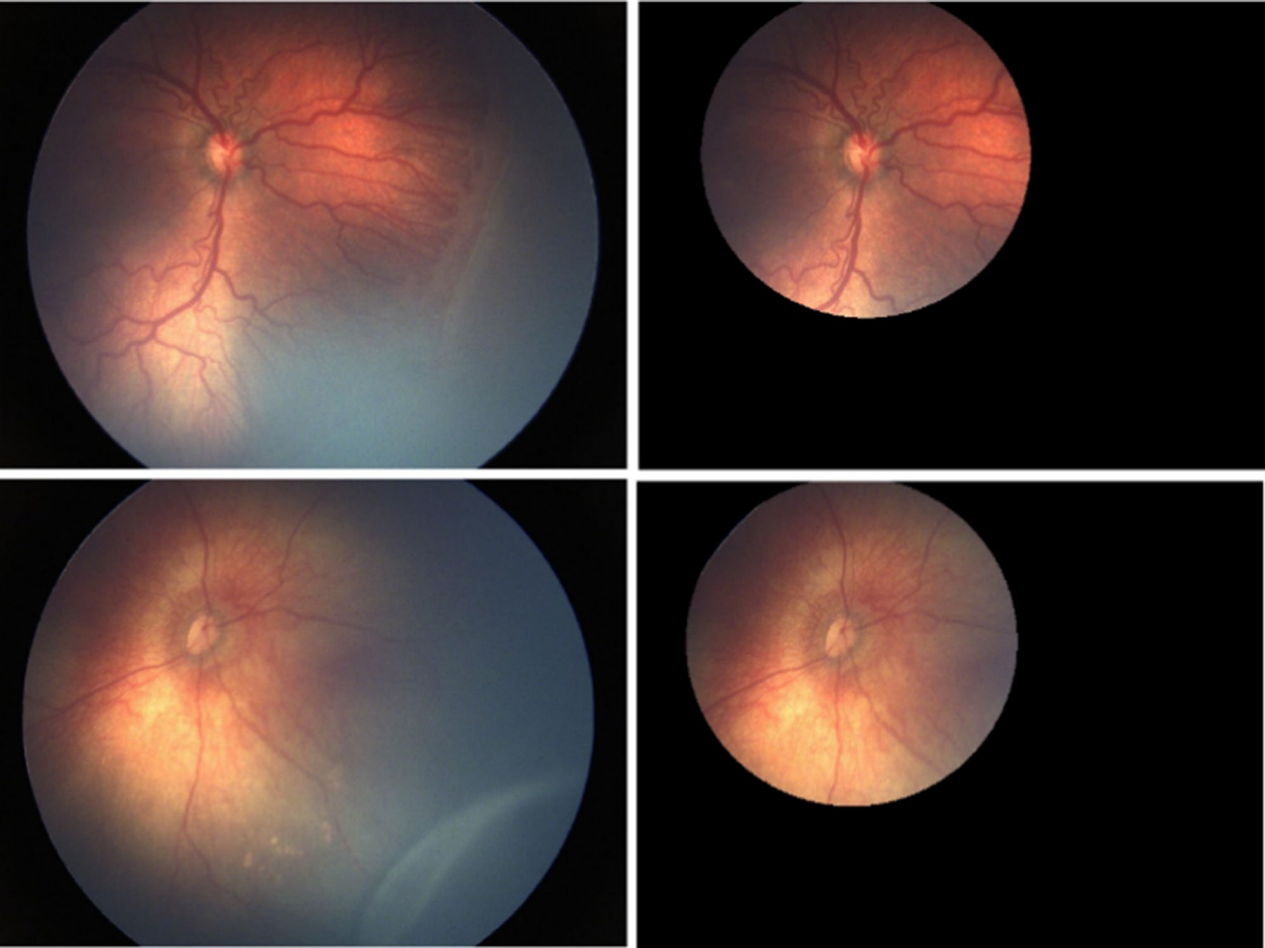
Two sample images and their corresponding cropped versions. Top row illustrates severe Plus disease characterized by venule dilation and arterioles tortuosity. The bottom row shows a non-Plus condition with a staged ROP characterized by a ridge line in the peripheral retina

Following the initial automated cropping, an expert conducted a final review of the cropped versions and rectified any potential flaws in the automated OD detection and cropping routines. Finally, none of the 3DD versions of the images included a ridge or demarcation line. Figure 1 illustrates an image and its corresponding 3DD version.

#### Grading routine

Experts independently classified both the entire-view images and their associated 3DD versions. To evaluate Plus disease, we utilized the International Classification of Retinopathy of Prematurity (ICROP3- Chiang et al. 2021) [11], which describes the characteristics of dilation and tortuosity of retinal vessels in zone 1 for determining plus disease. Moreover, in ICROP3, pre-plus disease is characterized by abnormal vascular dilation and/or tortuosity that does not meet the criteria for plus disease. Nevertheless, acknowledging that retinal vascular alterations in ROP indicate a continuous spectrum ranging from normal to pre-Plus to plus disease (as mentioned in the Chang et al. 2021), for a more precise classification of Plus, we requested experts to classify Plus images into three specific grades of Plus1, Plus2, and Plus3, according to the intensity of vessel tortuosity and dilation. Therefore, all images were randomly shuffled into five categories of disease severity including “normal”, “pre-Plus”, “Plus1”, “Plus2”, and “Plus3”. In a recently published study, we reported that how breaking down the individual “Plus” level into more specific levels of severity helps clinicians to better differentiate disease severity [13]. Accordingly, the images were assigned integer numerical scores of 1 to 5 based on the grades given by the experts. Images deemed unassessable by an expert due to insufficient evidence, were assigned a severity score of 0. Of note, if more than half of the experts deemed an image not assessable, it was excluded from the analysis. A reference standard diagnosis was established for each image based on the majority of the grades of the five experts. When there were discordant grades among the experts, a consensus meeting was held with all of the five experts to determine the final diagnosis. To minimize any biases in the grading process, an online interface was created to randomly display the entire-view and 3DD images to the experts, collect their grades, and create a database of labeled images.

### Outcomes of interest

Outcomes of interest included: (1) inter-expert reliability —for both entire-view and 3DD images, (2) impact of FOV on grading —comparison of the grades assigned to entire-view images versus 3DD and the direction of grade shifts, and (3) expert-specific grading patterns —analysis of individual expert’s grading patterns for entire-view and 3DD images to identify expert-specific susceptibility to variations in the FOV.

### Statistical analysis

Inter-class and intra-class correlation coefficients were calculated to assess data reliability. Grades from the entire-view images were compared to those from the 3DD versions to determine if there was a significant directional shift (e.g., less severe or more severe). For a detailed analysis, changes across severity levels were also examined by categorizing each grade as upscaled, downscaled, or unchanged. This analysis revealed how viewing the 3DD format influenced each Plus severity grading.

Statistical analysis was conducted using R version 3.3.1. To evaluate the reliability of the grading data, Cronbach’s alpha was calculated for both datasets of entire-view and 3DD images separately [14]. Prior to calculating the Cronbach’s alpha, a listwise deletion was applied to the data, i.e. excluding images recognized as unassessable by any of the experts from the datasets. A paired sample t-test (or Wilcoxon signed-rank test if data were non-normally distributed) was conducted to compare grades given to the entire-view images and their 3DD counterparts, determining if there was a significant directional shift in grading.

## Results

### Overall results of the reliability analysis

Seventy-four 3DD and 77 entire-view ROP images were included for grading process. Table 1 shows the number and percentage of images excluded from each of the datasets.

**Table 1:** This table shows the number of images included and excluded from the analysis due to listwise deletion and the corresponding cronbach’s alpha values for the “Entire” and “3DD” datasets

Datasets		Number (%)	Cronbach’s alpha	Number of items (Plus grades)
Entire	Valid	77 (84.6)	0.933	5 (from 1–5)
	Excluded	14 (15.4)		
	Total	91(100)		
3DD	Valid	74 (81.3)	0.942	5 (from 1–5)
	Excluded	17 (18.7)		
	Total	91 (100)		

In the 5-level grading system, Cronbach’s alpha values of 0.933 and 0.942 for the Entire and 3DD datasets, respectively, show an acceptable level of consistency in the grades given by the experts (Table 1). Both Cronbach’s alpha values (0.933 and 0.942) indicate high internal consistency reliability for the grades within each of the datasets with a slightly higher consistency among experts when assessing 3DD versions (0.942 > 0.933).

### Score shifts across field of views

**Fig. 2:**
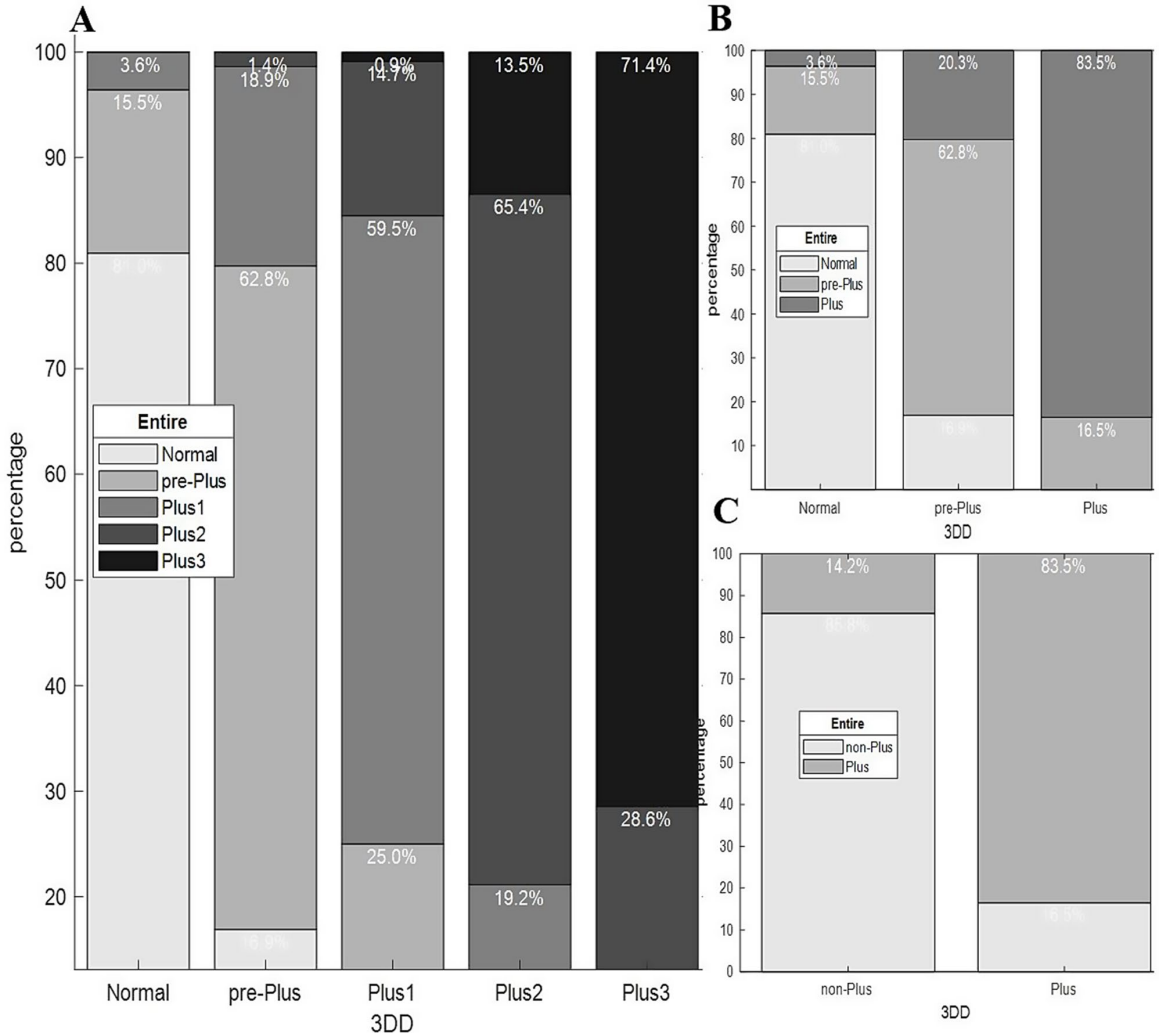
Impact of FOV on the assessment of Plus disease severity. These stacked bar charts show how the experts’ grades for entire images have changed (shifted) compared to the grades they gave to 3DD versions of the images. Each stacked bar illustrates the proportions of the grades at a specific severity level given to 3DD images that migrated to other levels when evaluating entire-view images. A, B, and C show grade shifts in 5-level, 3-level, and 2-level grading systems respectively. *Abbreviations*: 3DD: 3-disk-diameter, FOV: field of view

**Fig. 3:**
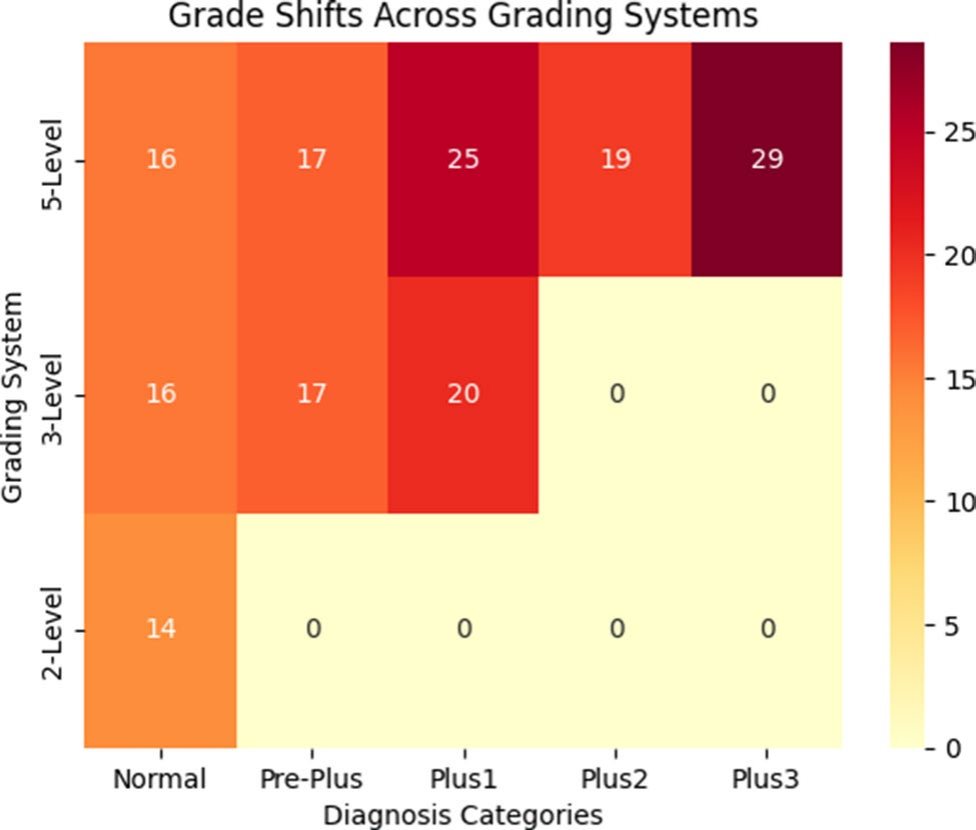
Grade Shifts Across Grading Systems. This heatmap illustrates the percentage of diagnostic shifts between entire-view and 3DD images across different grading systems (5-level, 3-level, and 2-level). Darker shades indicate higher percentages of shifts, highlighting the impact of field of view (FOV) on diagnostic consistency. The 5-level grading system exhibited the most pronounced shifts, particularly in the Plus1 and Plus3 categories

We compared grades given to the entire images and their 3DD versions to see if there was a significant shift in the grades towards a specific direction i.e. less severe or more severe. Figure 2 illustrates the percentages of the grades given to the 3DD that migrated to the other levels of severity when assessing the entire image. For instance, the left-most column in Fig. 2-A indicates that of all the normal grades assigned to 3DD images, 81% remained normal, while 15.5% upscaled to pre-Plus and 3.6% upscaled to Plus1 when evaluating entire-view images. There was a notable shift in the grades when evaluating the entire-view images compared to the 3DD versions across different grading systems (Figs. 2 and 3).

When assessing entire-view images compared to the 3DD versions, the following shifts were observed across different grading systems:

**1) 5-level grading system (Fig.** 2 **, A):**

- **Plus 3**: 28.6% of the diagnoses downscaled to Plus 2.
- **Plus 2**: 19.2% downscaled to Plus 1 and 13.5% upscaled to Plus 3.
- **Plus 1**: 25.0% upscaled to Plus 2, and 0.9% downscaled to pre-Plus.
- **Pre-Plus**: 16.9% downscaled to Normal, 18.9% upscaled to Plus 1, and 1.4% upscaled to Plus 2.
- **Normal**: 15.5% upscaled to pre-Plus, and 3.6% upscaled to Plus 1.

**2) 3-level grading system (Fig.** 2 **, B):**

- **Plus**: 16.5% of the diagnoses downscaled to pre-Plus when comparing entire-view to 3DD assessments.
- **Pre-Plus**: 16.9% downscaled to Normal, and 20.3% upscaled to Plus.
- **Normal**: 15.5% upscaled to pre-Plus, 3.5% upscaled to Plus.

**3) 2-level grading system (Fig.** 2 **, C):**

- **Plus**: 16.5% of the diagnoses downscaled to non-Plus.
- **Non-Plus**: 14.2% upscaled to Plus.

### Expert-specific gradings

**Fig. 4:**
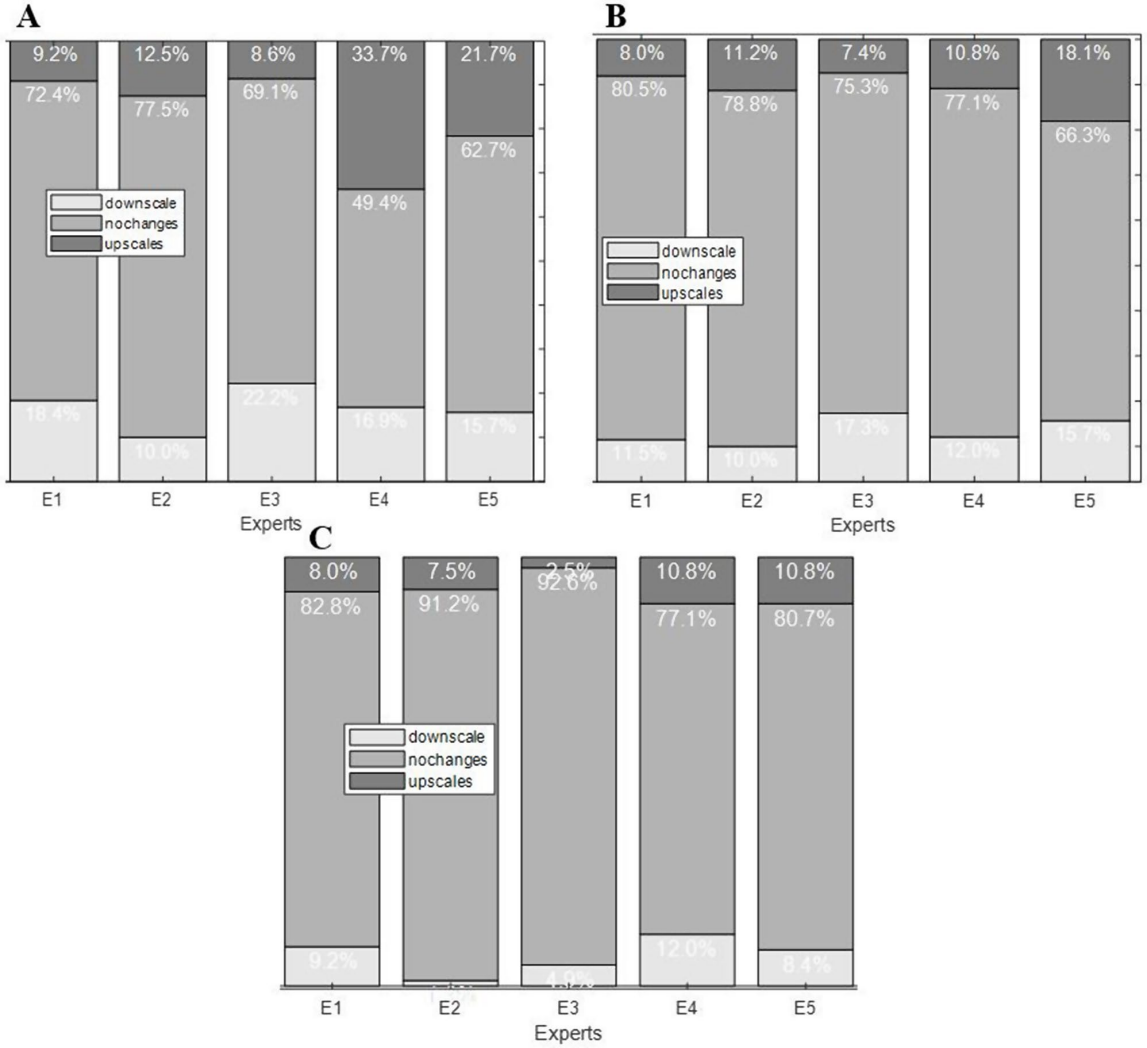
Grade shifts made by experts based on their 3DD and entire-view assessments in each of the grading systems. Each stacked bar illustrates the proportions of grades that were upscaled, downscaled, or stayed the same when an expert evaluated entire-view images versus when assessing 3DD images. A, B, and C show grade shifts made by experts in 5-level, 3-level, and 2-level grading systems respectively. *Abbreviations*: 3DD: 3-disk-diameter

Figures 4-A, -B, and -C show the distributions of the grades for each of the experts (E) based on two, three, and five-level grading systems, respectively. For example, column E5 in Fig, 3-A shows that in a five-level grading system, of all the grades assigned to 3DD images by the Expert 5, 62.7% remined intact, while 21.7% upscaled to more severe levels and 15.7% downscaled to less severe level when evaluating entire-view.

**5-level grading system (**Fig. 4, A**)**:

- **E4**: Showed 33.7% of their 3DD assessments being upscaled and 21.7% being downscaled when compared to their entire-view assessments.
- **E5**: Exhibited with 16.9% of their 3DD assessments upscaled and 15.7% downscaled.
- **E1**,** E2**,** and E3**: Demonstrated more modest shifts in their assessments.

**3-level grading system (**Fig. 4, B**)**:

- **E4**: Showed the most significant shifts, with 18.1% of their 3DD assessments upscaled and 10.8% downscaled.
- **E5**: Also exhibited substantial shifts, with 11.2% of their 3DD assessments upscaled and 7.4% downscaled.
- **E1**,** E2**,** and E3**: Demonstrated more consistent grading patterns across 3DD and entire-view evaluations.

**2-level grading system (**Fig. 4, C**)**:

- **E4**: Showed the most significant shifts, with 10.8% of their 3DD assessments upscaled and 8.0% downscaled.
- **E5**: Exhibited 7.5% of their 3DD assessments upscaled and 7.5% downscaled.
- **E1**,** E2**,** and E3**: Demonstrated more consistent grading patterns across 3DD and entire-view assessments.

**Fig. 5:**
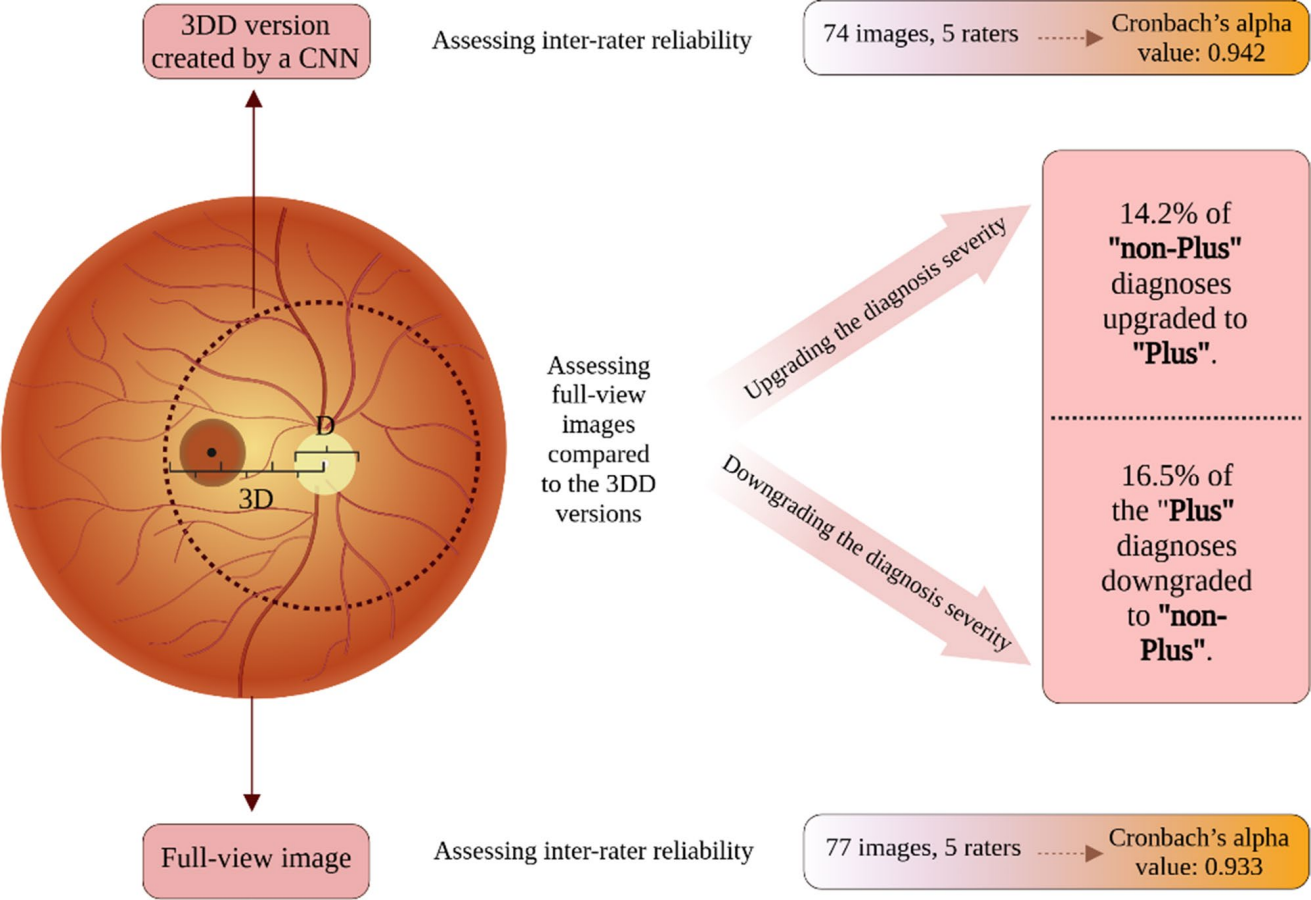
A summary of the study design and results. *Abbreviations*: CNN: convolutional neural network, D: optic disc diameter, 3DD: 3-disk-diameter. Created in https://BioRender.com

Figure 5 Shows a summary of the study design and results.

## Discussion

This study analyzed 77 entire-view and 74 cropped 3DD retinal images from infants with ROP. The images were categorized into different severity groups and graded by five ROP experts. Reliability analysis indicated slightly higher inter-expert consistency for 3DD images compared to entire-view images. Notable diagnostic shifts were observed when comparing entire-view and 3DD images (when only part of zone 1 is included), more noticeable within the 5-level grading system. Additionally, some experts demonstrated greater susceptibility to variation in the FOV. The findings underscore the influence of diagnostic variations/biases in ROP evaluation, which can be impacted by FOV. It, therefore, highlights the need for standardized imaging protocols and the implementation of AI into diagnostic steps to minimize variability and subjectivity in diagnosis [13, 15].

Although there is an updated published consensus on the classification of ROP [11], subjectivity in the diagnosis of Plus disease may still happen, as evident by the high level of diagnostic sifts when the FOV was changed in this investigation. Inconsistent identification of Plus disease can lead to diagnostic errors, such as downscaling the severity, resulting in undertreatment and failure to address sight-threatening conditions, or upscaling, leading to overtreatment and exposing patients to unnecessary adverse effects. Notably, even though this study involved experienced ROP experts, some were more influenced by changes in FOV, underscoring the critical role of FOV in diagnostic accuracy. Apart from the initial disease diagnosis, disagreements can also affect evaluations of treatment response, which is a crucial step in the process of disease treatment and monitoring. Taken together, these highlight the need to incorporate FOV considerations into clinical guidelines to minimize variability and improve diagnostic consistency.

The findings of this study align with previous research highlighting the complexity of Plus disease diagnosis. Hewing et al. demonstrated that experts exhibit variability in their reasoning processes, focusing on different retinal features and interpreting them differently [16]. Our study further supports this by showing that FOV can significantly influence expert assessments. By restricting the FOV to a 3DD area, we may have inadvertently encouraged experts to focus more on specific vascular characteristics (arterial tortuosity and venous dilation) as suggested by Hewing et al., potentially leading to increased consistency in their assessments as reflected by the slightly higher Cronbach’s alpha for the 3DD dataset.

The findings of the current study are consistent with previous research highlighting the challenges of inter-observer agreement in ROP classification. Campbell et al. demonstrated that significant discrepancies exist among experts in ROP classification, particularly regarding stage and Plus disease [17]. Our study further supports these findings by showing that FOV variations can significantly influence the diagnosis of Plus disease, potentially contributing to the observed discrepancies. By restricting the FOV to a 3DD area, we may have inadvertently introduced a source of variability in Plus disease diagnosis, which could contribute to the overall inter-observer variability observed in previous studies. These findings underscore the importance of standardizing both image acquisition and analysis protocols, including FOV considerations, to improve the consistency and reliability of ROP diagnosis.

Our results contradict the findings of Rao et al. (2012) [9]. They used 15 wide-angle images from ROP patients which were then cropped to create two further image groups including medium-angle (40–50°) and narrow-angle (20–30°). Finally, 45 images were assessed by 13 ROP experts using a (1) 2-level (Plus, non-Plus) and (2) 3-level (Plus, pre-Plus, neither) classification. In both 2-level and 3-level classifications, the level of agreement by experts was significantly higher when examining wide-angle images. Hence, the authors concluded that a wider field of view resulted in more consistency in the diagnosis and suggested that taking into account peripheral findings may also assist the experts in the accurate diagnosis [9]. However, other factors, such as the difference in the size of the cropped images and included zones between our study and the study by Rao et al., might account for this discrepancy. Importantly, high levels of agreement don’t necessarily suggest that the diagnoses are correct, particularly in small sample sizes. Hence, future studies should include a larger number of retinal images to more accurately evaluate the impact of FOV on Plus diagnosis.

### Strengths and limitations

This study has several strengths. First, we used three grading systems (two-, three-, and five-level grading systems), which enabled us to have a broader evaluation of the effect of FOV on diagnostic consistency across different disease stages. Secondly, we implemented a CNN model to automatically create the 3DD images, significantly reducing the time burden on the investigators. Finally, we evaluated individual experts’ shifts in diagnoses across the two image formats to understand the interpersonal variability in susceptibility to FOV variations.

The results of this investigation should be interpreted in light of its drawbacks, with the most prominent being the retrospective nature of the study and limited number of experts and images and retrospective nature of the study primarily due to the limitations set by the inclusion criteria. Furthermore, conducting additional grading sessions to reiterate the experts’ assessments for each image version might yield a more dependable metric for inter-expert variability and help in reaching a more precise conclusion regarding the influence of field of view.

## Conclusions

This study investigated the impact of FOV on the diagnosis of Plus disease in ROP. Our findings demonstrate that FOV can significantly influence expert assessments, leading to variations in Plus disease diagnosis. While restricting the FOV to a 3-disc diameter (3DD) area around the OD resulted in slightly higher inter-expert reliability (Cronbach’s alpha of 0.942 vs. 0.933 for entire-view images), it also led to notable shifts in diagnoses. We observed a substantial proportion of images being upscaled or downscaled in severity when comparing entire-view and 3DD assessments. These shifts were more pronounced in the 5-level grading system, suggesting that including or excluding peripheral retinal features can impact expert judgments. Furthermore, our analysis revealed that certain experts were more susceptible to FOV variations than others, highlighting the importance of considering individual expert tendencies. Further research is required to identify additional factors contributing to diagnostic variability among experts and to develop guidelines that improve the accuracy and consistency of ROP diagnosis.

## Data Availability

The datasets developed and/or analyzed during the current study are available from the corresponding author upon reasonable request.
